# Effect of Educational Handouts With Standard Therapy Versus Standard Therapy Alone on Compliance With Oral Iron Supplementation in Antenatal Women With Iron Deficiency Anemia: A Randomized Controlled Trial

**DOI:** 10.7759/cureus.39508

**Published:** 2023-05-25

**Authors:** Anushree Shetty, Anuja Bhalerao, Anjali Kawathalkar, Charmy Vashi

**Affiliations:** 1 Obstetrics and Gynaecology, N.K.P. Salve Institute of Medical Sciences & Research Centre and Lata Mangeshkar Hospital, Nagpur, IND; 2 Obstetrics and Gynaecology, N.K.P. Salve Institute of Medical Sciences & Research Centre, Nagpur, IND

**Keywords:** iron deficiency anemia, antenatal women, oral iron supplementation, compliance, standard therapy, educational handouts

## Abstract

Background

Iron requirements rise dramatically throughout the second and third trimesters of pregnancy. Pregnant women are more susceptible to anemia because their need for iron increases during pregnancy, which is difficult to achieve through diet alone.

Methodology

A randomized controlled trial (non-blinded and parallel group) was undertaken with the recruitment of 174 women. However, 35 women were lost to follow-up, and the study was ultimately completed with 139 participants distributing 68 women in Group A (intervention group) and 71 women in Group B (non-interventional group). Educational handouts were explained to the participants with iron supplements in Group A and only supplements were given to Group B, and the participants were followed up till three months before the recruitment period. Compliance with iron supplementation and a rise in hemoglobin were noted.

Results

In this study, maximum women were in the 22-30 years age group and were almost evenly distributed with respect to parity with no statistically significant difference in the groups. All the participants were started with oral iron therapy. No additional parenteral iron therapy was given. Women in Group A showed good compliance for iron supplementation than those in Group B. It was determined that this difference was statistically insignificant (>0.05). In the majority of women, the reason for poor compliance was frustration to follow oral iron therapy daily (52.3% in Group A and 21.7% in Group B). There were other reasons like forgetfulness, heartburn, vomiting, constipation, and nausea as the reason for poor compliance. The hemoglobin levels were compared at the recruitment and a mean rise in hemoglobin levels was noted in groups A and B at the follow-up period after three months. There was a greater mean rise in hemoglobin concentration in Group A (1.28) than in Group B (0.63), which was statistically insignificant (>0.05).

Conclusion

The current study found that among pregnant women with iron-deficient anemia, instructional handouts did not promote compliance with oral iron treatment. The main reasons for low compliance were frustration with taking the oral drug, followed by forgetfulness, heartburn, vomiting, constipation, and nausea. In pregnant females with anemia brought on by iron deficiency, educational handouts did not enhance hemoglobin status.

## Introduction

A common public health issue impacting 1.6 billion people worldwide is anemia [1]. Loss of productivity due to anemia causes a substantial economic burden [2]. In India, there are 50.4% more anemic pregnant women than normal, which is the most common hematological disease. Evidence suggests that the need for iron increases significantly throughout the trimesters of pregnancy, mainly the second and third trimesters [3]. During pregnancy, because of the rise in iron requirements and the difficulty of meeting these requirements through diet alone, pregnant women are particularly vulnerable to anemia [4]. Despite significant health initiatives, the incidence of iron deficiency anemia (IDA) has led to 20% maternal fatalities in India [5]. Maternal anemia is thought to increase the chance of an unfavorable pregnancy outcome and pose a threat to both the mother's and the fetus' lives [6]. Maternal anemia in pregnancy can lead to an increase in fetal wasting, stillbirth, low birth weight, and perinatal mortality [7].

A key reason for the persistently high rate of IDA in pregnant women in India is decreased compliance with iron supplementation orally [8,9]. The unfavorable effects of ingesting iron pills, which are related to the quantity [10] and form of the tablets, as well as perhaps to the frequency and duration of their administration, have an impact on compliance. One of the crucial techniques suggested by the WHO to prevent, regulate, and manage anemia through health education programs is the modification in behavior [11]. Pregnant women's awareness, compliance, and anemia prevalence were significantly improved thanks to health education programs [12].

The patients visiting tertiary care centers are invariably from different socio-economic strata of society with less health awareness and low educational status, this may lead to inadequate knowledge in the patient, which may change patients’ attitude and show a lack of motivation regarding taking oral iron supplementation. Also, the present scenario shows a scarcity of studies focusing on improving oral iron supplementation in our population in the Maharashtra region. To overcome this practical difficulty, the current study was designed to evaluate the effect of educational handouts with standard therapy and to assess the cause of poor compliance with oral iron therapy in antenatal women with IDA-complicating pregnancies.

## Materials and methods

This is a non-blinded, parallel-group, randomized controlled trial (RCT) conducted at the department of obstetrics and gynecology of a tertiary care hospital from January 2021 to December 2022 after getting approval from the Institutional Ethics Committee, N.K.P. Salve Institute of Medical Sciences and Research Centre (reference number: 97/2021). Informed consent was taken and a total of 174 women were included in this study and randomized equally into two groups by convenience sampling technique. The inclusion criteria included antenatal women with gestational age between 14 and 24 weeks of pregnancy with a hemoglobin level between 9 and 10.9 gm/dl with AA pattern on hemoglobin electrophoresis, willing to communicate telephonically, and able to read in their vernacular language (Hindi, Marathi, or English) or should have a relative accompanying them who can read and explain the educational handouts. The exclusion criteria included those not responding to mobile phone calls on three consecutive days, having an abortion or pre-term delivery within three months of the study period, and having bleeding per vaginum during three months.

The method of hemoglobin estimation was the flow cytometry method. Hemoglobin was measured in gram percent. IDA was diagnosed on the basis of a complete blood count and peripheral smear report and hemoglobin electrophoresis showing an AA pattern. Hemoglobin electrophoresis was done to rule out hemoglobinopathies. Permuted block randomization was used with a block size of 6 to randomly allocate study participants into two groups, using a predetermined computer-generated random allocation plan. Recruited participants were randomized into two groups. After identifying the women to be recruited in the study as per the selection criteria by the principal investigator, the nurse in antenatal OPD was allotting the women randomly as per computer-generated numbers in the two groups as the allocation ratio was 1:1.

In Group A (the intervention group), standard oral iron therapy in addition to educational handouts in English, Hindi, and Marathi was provided. The participants were asked to read the handouts under the supervision of the principal investigator and if they could not read, the relative accompanying the participant was made to read it out. After the participant and relative had read the handout, the principal investigator answered the queries by the participant and relative regarding the handouts. Participants were instructed to follow the instructions written in the educational handout during their subsequent antenatal visits. In Group B (the control group), standard oral iron therapy alone for IDA was prescribed. All women were provided with iron (ferrous sulfate) tablets of 100 mg having 60 mg of elemental iron supplied free of cost by the government along with a folic acid tablet of 5 mg. Women were instructed to take iron tablets one hour before a main meal with a twice-a-day dosage and folic acid was advised to be taken once a day.

**Figure 1:**
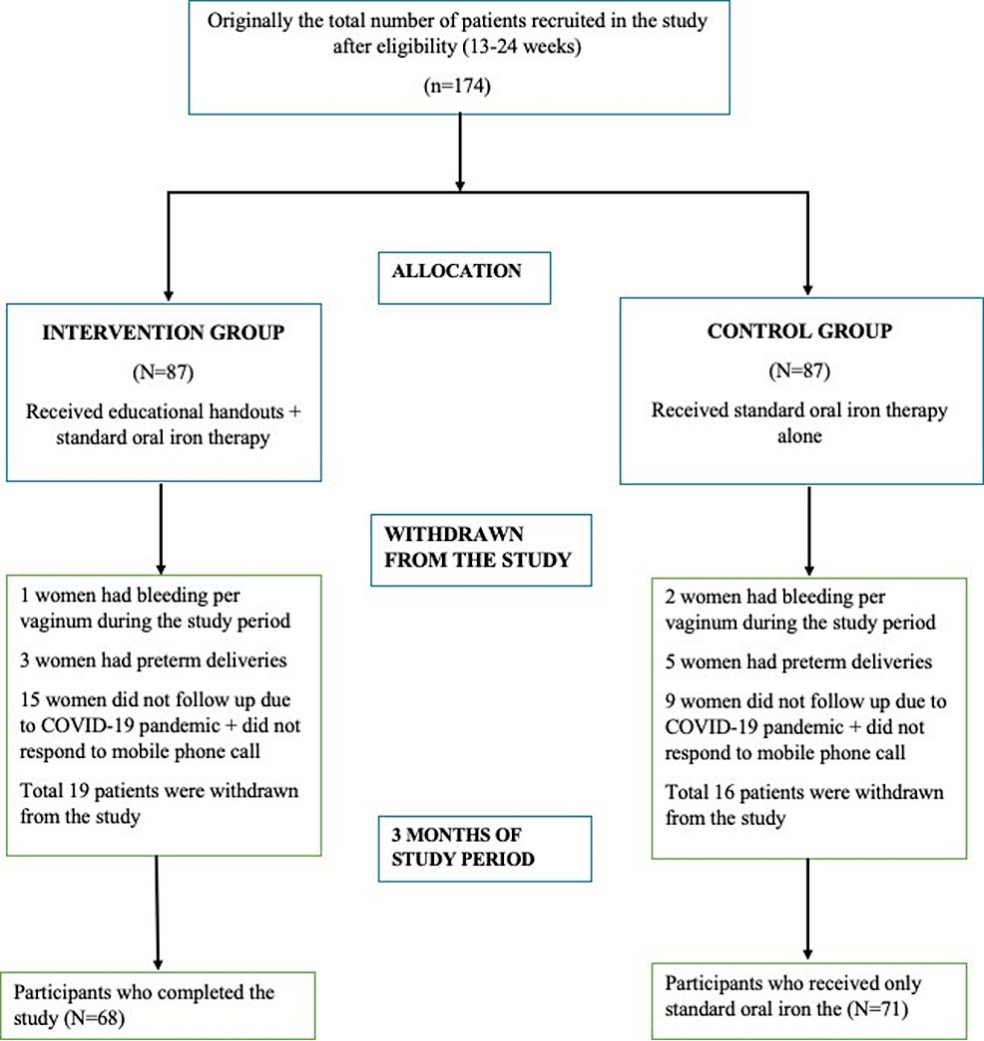
Study flowchart

Iron tablets were provided in a small plastic pouch with 60 tablets of iron and 30 tablets of 5 mg of folic acid provided for one month. All women were instructed to bring back the pouch with the remaining tablets at every visit. If follow-up was not possible in the outpatient department due to the coronavirus disease 2019 (COVID-19) pandemic, then the patient was asked on the phone at her convenient time about the number of iron tablets consumed that month. If patients failed to consume the tablets, then patients were asked about the reasons behind their noncompliance. One-month medication was provided and the same dosage was repeated at monthly antenatal visits. If the participant was not able to visit the outpatient department, then they were asked to take the tablets from a nearby government hospital, and follow-up was taken on the phone. In case of inability to contact by mobile phone, repeat phone calls were given on the next three consecutive days after that they were considered as lost to follow up. Women in both groups were followed up to three months after their recruitment, as shown in Figure 1. Compliance [11] was ascertained by the pill count method at every antenatal check-up or via phone calls. The number of remaining tablets in the pouch was counted by the researcher when the women were able to come for follow-up antenatal visits. Whenever the follow-up was taken with the help of mobile phone calls, the women were asked to count the remaining tablets in the pouch at the end of each month and the number of consumed tablets was noted in the case record form. The participants' hemoglobin was assessed twice, the first time when the patients were recruited and the second time at the end of three months.

SPSS version 20 (IBM Corp., Armonk, NY) was used for statistical analysis. Quantitative data are presented using the mean and standard deviation. An unpaired t-test was used to compare the research groups in accordance with the results of the normality test. To present the qualitative data, a frequency and percentage table was employed. To determine whether there was any correlation between the research groups, the Student’s t-test, Fisher's test, and chi-square test were applied. Significant results were those with a p-value of 0.05 or less. The graphical representation was done in Microsoft Excel 2010 (Microsoft Corporation, Redmond, WA).

## Results

A non-blinded, randomized control trial with 139 patients was undertaken to analyze the impact of educational handouts combined with standard therapy on enhancing adherence to oral iron tablet supplementation in female antepartum patients with IDA. Patients were split into two groups. A total of 35 patients were withdrawn from the study on account of per vaginum bleeding during the three-month study period (one patient from Group A and two from Group B), preterm deliveries (three patients from Group A and five patients from Group B), discontinuity due to COVID-19 pandemic, and no response to the mobile phone calls (15 from Group A and nine patients from Group B). The remaining patients in the study during follow-up in Group A were 68 patients and in Group B were 71 patients. The loss to follow-up in this study was 20.1%. It was more than 10% due to the COVID-19 pandemic; a lesser number of patients visited the hospital and did not follow up.

In Group A, 47.2% were in the age group of 26-30 years, followed by 29.9% of patients in 21-25 years, 17.2% of patients in >30 years, and 5.7% of patients in the age group of 18-20 years, deriving the mean age of patients as 26.89 ± 4.34 years. However, in Group B, 52.9% were in the age group of 21-25 years, followed by 25.3% of patients in 26-30 years, 19.5% of patients in >30 years, and 2.3% of patients in the age group of 18-20 years, deriving the mean age of patients as 26.18 ± 4.46 years, with no significant difference between the groups as per the Student's t-test (p > 0.05). The distribution according to the parity is shown in Table 1. The difference was statistically not significant as per the chi-square test (p > 0.05).

**Table 1:** Distribution of patients according to parity

Parity	Group A (intervention)		Group B (control)		p-value
	N	%	N	%	
Nulliparous	35	50.6%	38	54.1%	>0.05
Multiparous	33	49.4%	33	45.9%	
Total	68	100%	71	100%	

**Figure 2:**
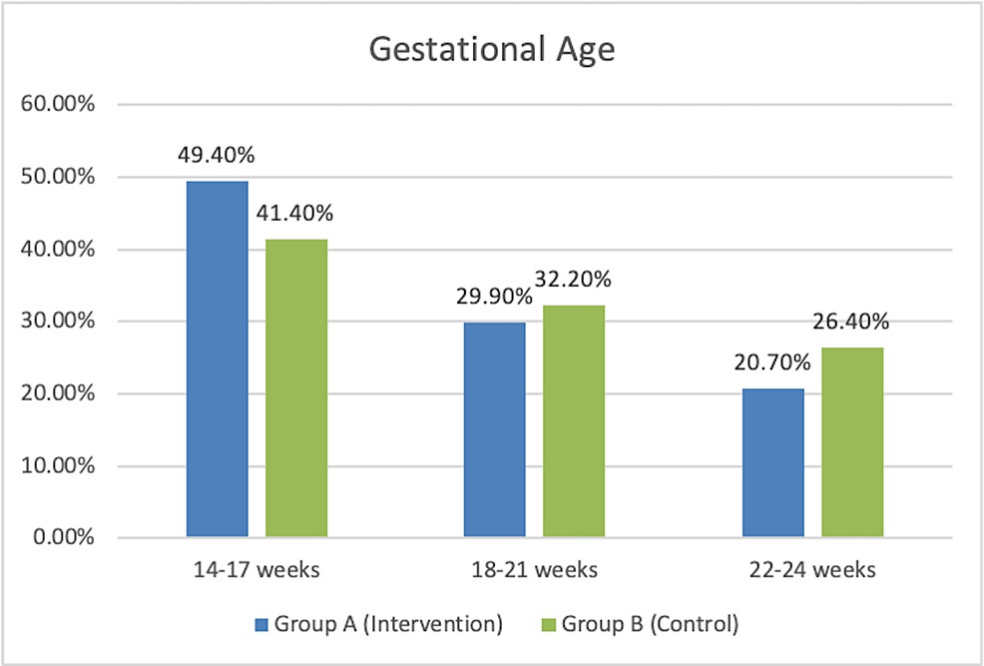
Distribution of patients according to gestational age at the time of recruitment

The distribution of gestational age at the time of recruitment among Groups A and B is shown in Figure 2. There was no significant difference between the groups as per the chi-square test (p > 0.05).

**Figure 3:**
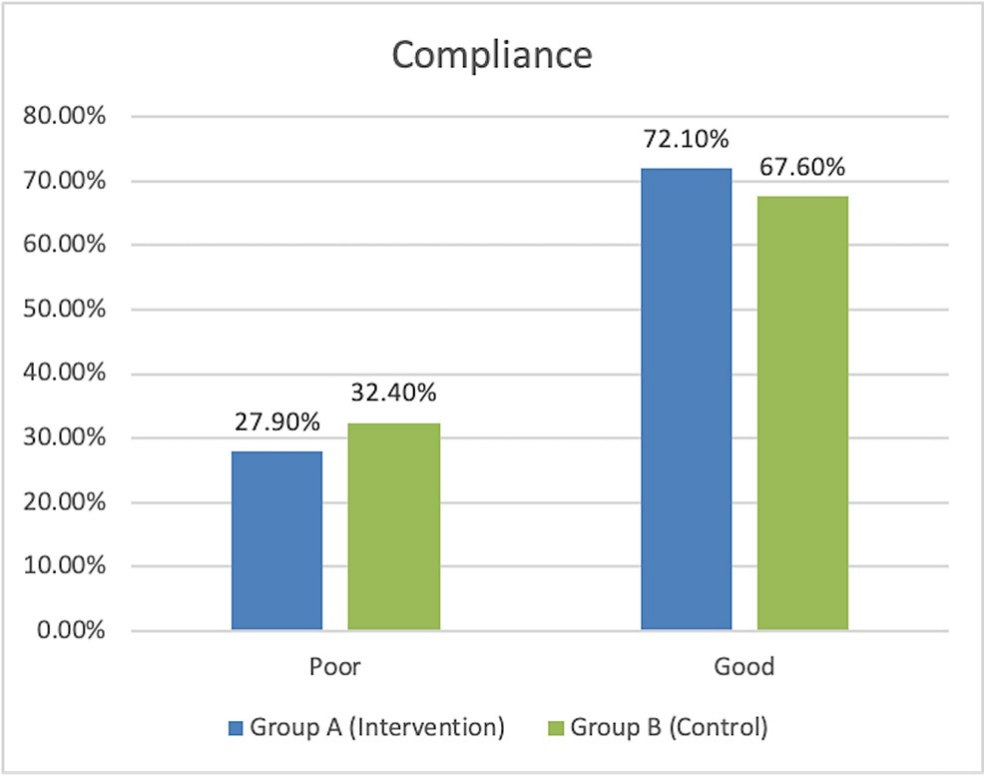
Distribution of patients according to the poor and good compliance

The distribution of patients as per compliance showed no significant difference between the groups as per the chi-square test (p > 0.05), as shown in Figure 3. The majority of the patients in Groups A and B had compliance between 65% and 84%, and there were no participants who had compliance below 45% in both groups, as shown in Table 2.

**Table 2:** Distribution of patients according to compliance

Compliance	Group A (intervention)		Group B (control)		p-value
	N	%	N	%	
<25%	-	-	-	-	>0.05
25-44%	-	-	-	-	
45-64%	19	27.9%	23	32.4%	
65-84%	43	64%	40	56.3%	
85% and more	6	8.8%	8	11.2%	
Total	68	100%	71	100%	

The analysis of the reason for poor compliance was measured for Groups A and B. Majority of the patients in Group A (52.3%) gave frustration as the main reason for poor compliance followed by forgetfulness (21.2%), heartburn (10.6%), vomiting (10.6%) and constipation (5.3%). Five (21.7%) patients in Group B gave frustration as the main reason for poor compliance while four (17.4%) patients each gave forgetfulness, heartburn, constipation, nausea, and vomiting as the reason for poor compliance. Two (8.7%) patients mentioned gastritis. The mean hemoglobin levels at the time of recruitment and at three months follow-up were compared between the groups and no statistical significance was found as per the Student's t-test, as shown in Table 3. However, the change in mean hemoglobin was more in the intervention group after three months of study but the change was not statistically significant (>0.05).

**Table 3:** Distribution of patients according to hemoglobin levels at the time of recruitment and at three-month follow-up

Hemoglobin (gm %)	Group A (intervention)		Group B (control)		p-value
	Mean	SD	Mean	SD	
Time of recruitment	9.74	0.66	9.81	0.69	>0.05
Three-month follow-up	10.75	0.93	10.44	0.96	>0.05

## Discussion

A total of 139 women were recruited in the current non-blinding, parallel-group, randomized controlled trial. With a mean age of 26.8 in Group A (intervention group) and 26.18 in Group B (non-interventional group). The majority of the prenatal women in the current study were between the ages of 25 and 30 years. However, Khorshid et al. [13], Byamugisha et al. [14], Ahamed et al. [15], Kaundal et al. [16], Sunuwar et al. [12], Abujilban et al. [17], Srivastava et al. [18], Nahrisah et al. [19], Bumrungpert et al. [20], Sontakke et al. [21], Mithra et al. [22], Dutta et al. [23], Kassa et al. [24], and Boti et al. [25] reported mean ages ranging from 22-30 years of age. Similar to a study done by Sontakke et al. [21], pregnant women in the present study were equally divided according to their parity status as nulliparous and multiparous in intervention and non-interventional groups. There were fewer multiparous women than nulliparous in previous studies undertaken by Wiradnyani et al. [26], Byamugisha et al. [14], Nahrisah et al. [19], and Kassa et al. [24]. Comparatively, only a few other studies by Boti et al. [25] and Khorshid et al. [13] included more nulliparous women.

As anemia is so prevalent and treatable, various interventions were undertaken in an attempt to improve compliance with oral iron supplementation and hemoglobin. The present study used educational handouts along with standard oral iron therapy to improve compliance and hemoglobin. The educational handouts were given to the patients in a language best understood by them, and knowledge regarding oral iron supplementation was explained to the patients. Doubts of the patients were cleared, and if they were illiterate, their relatives accompanying them were explained, and they were told to read the handouts frequently, so as to enhance the importance of iron supplementation in antenatal women. Khorshid et al. [13], Byamugisha et al. [14], Ahamed et al. [15], Kaundal et al. [16], Sunuwar et al. [12], Abujilban et al. [17], and Srivastava et al. [18] reported the gestational age between 14 and 24 weeks (second trimester of pregnancy), supporting the findings of the present study, except a study conducted by Nahrisah et al. [19] who included antenatal women from all gestational ages. The present study included antenatal women with hemoglobin between 9 and 10.9 gm/dl, having AA pattern on hemoglobin electrophoresis, who were capable of reading in Hindi, Marathi, or English or at least have a relative accompanying them who can read. Patients who were intolerant to oral iron were excluded and antenatal women with bleeding per vaginum and who had preterm delivery during the study period were excluded. The other studies conducted by Khorshid et al. [13], Kaundal et al. [16], and Bumrungpert et al. [20] used serum ferritin in their inclusion criteria. Few other studies conducted by Abujilban et al. [17] and Sontakke et al. [21] included women with mobile phones. Most of the studies excluded women's obstetrical complications like severe anemia, hematological disorder, bleeding in pregnancy, heart failure, and a few others.

Mithra et al. [22], Khorshid et al. [13], Dutta et al. [23], Wiradnyani et al. [26], Byamugisha et al. [14], Boti et al. [25], Ahamed et al. [15], Abujilban et al. [17], Kassa et al. [24], Srivastava et al. [18], Bumrungpert et al. [20], and Sontakke et al. [21] applied either compliance or adherence or pill count to assess the oral intake of iron tablets. Although these studies employed compliance, adherence, and pill count to gauge the results, there are no set criteria to distinguish between great and poor compliance with iron supplementation during pregnancy. The cutoff point in various studies differs as set by the researcher. Hence, this makes it difficult to compare various studies and their outcomes, as there is no uniformity.

The present study had an increase in compliance as compared to the control group, which did not receive the educational handouts. But the increase in compliance in the intervention group was not statistically significant. Khorshid et al. [13], Wiradnyani et al. [26], Abujilban et al. [17], Nahrisah et al. [19], and Sontakke et al. [21] found a positive effect on compliance with the association of different interventions. However, Byamugisha et al. [14] and Srivastava et al. [18] showed no statistical significance, supporting the findings of the present study. This study emphasized that various factors like literacy, that is patients who can read and write, had better compliance and factors like the interpersonal relationship between researcher and patient along with forgetfulness and active participation by patients were various factors that contributed to compliance, hence the effect on compliance with oral iron supplementation was multifactorial.

In the current study, the majority of women cited annoyance as the cause of their low compliance with daily therapy of oral iron. Apart from this, frustration, low education, constipation, vomiting, heartburn, and forgetfulness contributed to poor compliance. Nahrisah et al. [19] found forgetfulness as a major contributor to non-compliance. Many other reasons like cost, literacy, low knowledge of anemia, and interpersonal relations between the provider and antenatal women were some of the factors that contributed to compliance with oral iron supplementation, which need to be dealt with. Hemoglobin levels increased in the current study compared to the mean levels at recruitment, although neither the intervention group nor the control group experienced a statistically significant increase in hemoglobin levels. Khorshid et al. [13], Srivastava et al. [18], and Kaundal et al. [16] reported no major change in the mean hemoglobin levels contradicting the significant findings mentioned in the study by Byamugisha et al. [14]. The limitation of the study states that the availability of mobile phones limited the population from participating in the study.

## Conclusions

The current study concluded that the educational handouts do not increase compliance with oral supplementation of iron in pregnant women with IDA. The most common cause of poor compliance is frustration to consume the oral medication followed by forgetfulness, heartburn, vomiting, constipation, and nausea. Educational handouts do not improve the hemoglobin status in antenatal females with anemia caused due to deficiency of iron.

## Figures and Tables

**Figure 4:**
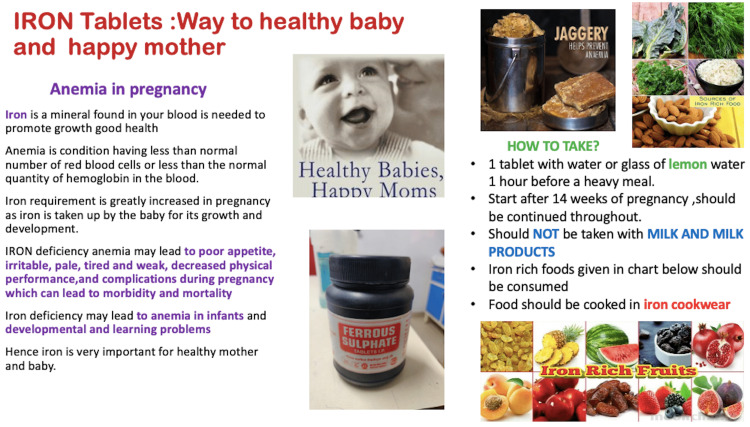
Educational handout in the English language

**Figure 5:**
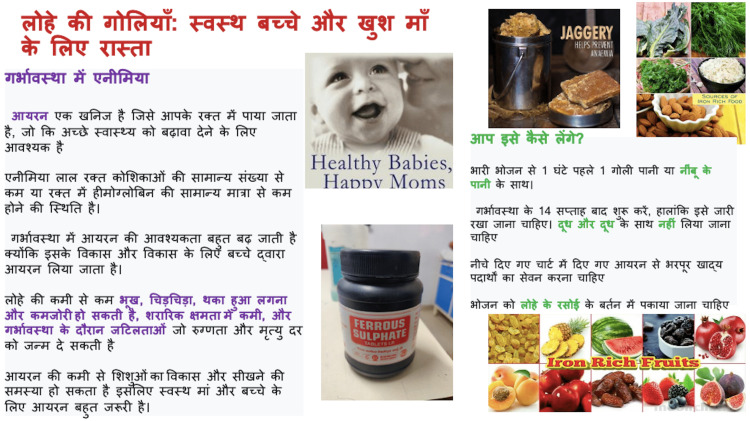
Educational handout in the Hindi language

**Figure 6:**
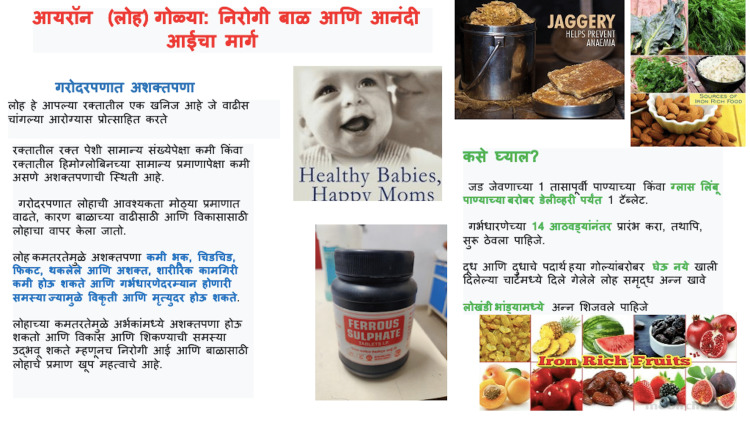
Educational handout in the Marathi language

## Appendices

The educational handouts used in Group A were in English (Figure 4), Hindi (Figure 5), and Marathi languages (Figure 6).

## References

[1] McLean E, Cogswell M, Egli I, Wojdyla D, de Benoist B (2009). Worldwide prevalence of anaemia, WHO Vitamin and Mineral Nutrition Information System, 1993-2005. Public Health Nutr.

[2] Haas JD, Brownlie T 4th (2001). Iron deficiency and reduced work capacity: a critical review of the research to determine a causal relationship. J Nutr.

[3] Moghaddam Tabrizi F, Barjasteh S (2015). Maternal hemoglobin levels during pregnancy and their association with birth weight of neonates. Iran J Ped Hematol Oncol.

[4] Reveiz L, Gyte GM, Cuervo LG, Casasbuenas A (2011). Treatments for iron-deficiency anaemia in pregnancy. Cochrane Database Syst Rev.

[5] Kapil U, Kapil R, Gupta A (2019). National Iron Plus Initiative: current status & future strategy. Indian J Med Res.

[6] Pappas G, Akhtar T, Gergen PJ, Hadden WC, Khan AQ (2001). Health status of the Pakistani population: a health profile and comparison with the United States. Am J Public Health.

[7] Kalaivani K (2009). Prevalence & consequences of anaemia in pregnancy. Indian J Med Res.

[8] Lacerte P, Pradipasen M, Temcharoen P, Imamee N, Vorapongsathorn T (2011). Determinants of adherence to iron/folate supplementation during pregnancy in two provinces in Cambodia. Asia Pac J Public Health.

[9] Mannar V, Gallego EB (2002). Iron fortification: country level experiences and lessons learned. J Nutr.

[10] Charoenlarp P, Dhanamitta S, Kaewvichit R (1988). A WHO collaborative study on iron supplementation in Burma and in Thailand. Am J Clin Nutr.

[11] Bora R, Sable C, Wolfson J, Boro K, Rao R (2014). Prevalence of anemia in pregnant women and its effect on neonatal outcomes in Northeast India. J Matern Fetal Neonatal Med.

[12] Sunuwar DR, Sangroula RK, Shakya NS, Yadav R, Chaudhary NK, Pradhan PM (2019). Effect of nutrition education on hemoglobin level in pregnant women: a quasi-experimental study. PLoS One.

[13] Khorshid MR, Afshari P, Abedi P (2014). The effect of SMS messaging on the compliance with iron supplementation among pregnant women in Iran: a randomized controlled trial. J Telemed Telecare.

[14] Byamugisha J, Adero N, Kiwanuka TS (2022). The effect of blister packaging iron and folate on adherence to medication and hemoglobin levels among pregnant women at National Referral Hospital antenatal clinics in a low to middle income country: a randomised controlled trial (the IFAd trial). BMC Pregnancy Childbirth.

[15] Ahamed F, Yadav K, Kant S, Saxena R, Bairwa M, Pandav CS (2018). Effect of directly observed oral iron supplementation during pregnancy on iron status in a rural population in Haryana: a randomized controlled trial. Indian J Public Health.

[16] Kaundal R, Bhatia P, Jain A (2020). Randomized controlled trial of twice-daily versus alternate-day oral iron therapy in the treatment of iron-deficiency anemia. Ann Hematol.

[17] Abujilban S, Hatamleh R, Al-Shuqerat S (2019). The impact of a planned health educational program on the compliance and knowledge of Jordanian pregnant women with anemia. Women Health.

[18] Srivastava R, Kant S, Singh AK, Saxena R, Yadav K, Pandav CS (2019). Effect of iron and folic acid tablet versus capsule formulation on treatment compliance and iron status among pregnant women: a randomized controlled trial. J Family Med Prim Care.

[19] Nahrisah P, Somrongthong R, Viriyautsahakul N, Viwattanakulvanid P, Plianbangchang S (2020). Effect of integrated pictorial handbook education and counseling on improving anemia status, knowledge, food intake, and iron tablet compliance among anemic pregnant women in Indonesia: a quasi-experimental study. J Multidiscip Healthc.

[20] Bumrungpert A, Pavadhgul P, Piromsawasdi T, Mozafari MR (2022). Efficacy and safety of ferrous bisglycinate and folinic acid in the control of iron deficiency in pregnant women: a randomized, controlled trial. Nutrients.

[21] Sontakke P, Dwidmuthe KS, Kawathalkar A, Bhalerao A (2022). Effect of mobile phone call reminders with standard therapy versus standard therapy alone on compliance with iron supplementation in antenatal women with iron deficiency anemia: a randomized controlled trial. Cureus.

[22] Mithra P, Unnikrishnan B, Rekha T (2014). Compliance with iron-folic acid (IFA) therapy among pregnant women in an urban area of south India. Afr Health Sci.

[23] Dutta AJ, Patel P, Bansal RK (2014). Compliance to iron supplementation among pregnant women: a cross sectional study in urban slum. Natl J Community Med.

[24] Kassa ZY, Awraris T, Daba AK, Tenaw Z (2019). Compliance with iron folic acid and associated factors among pregnant women through pill count in Hawassa city, South Ethiopia: a community based cross-sectional study. Reprod Health.

[25] Boti N, Bekele T, Godana W, Getahun E, Gebremeskel F, Tsegaye B, Oumer B (2018). Adherence to iron-folate supplementation and associated factors among pastoralist's pregnant women in Burji districts, Segen Area People's Zone, Southern Ethiopia: community-based cross-sectional study. Int J Reprod Med.

[26] Wiradnyani LA, Khusun H, Achadi EL, Ocviyanti D, Shankar AH (2016). Role of family support and women's knowledge on pregnancy-related risks in adherence to maternal iron-folic acid supplementation in Indonesia. Public Health Nutr.

